# Notes and News

**Published:** 1888-05-12

**Authors:** 


### NOTES AND NEWS.

Collected and Communicated.

Mr. Thomas Sanctuary, M.D., L.R.C.P., L.R.C.S., L.S.A., has been appointed to the new post of assistant medical officer to the London Skin Hospital.

Mr. and Mrs. Kendall visited the bazaar held on behalf of the new hospital in Holloway Road on Monday, and were enthusiastically received. In the course of the afternoon Mrs. Kendall gave two readings on behalf of the building fund to audiences which filled the large hall to overflowing in every part. The readings were listened to with appreciative delight, and Mrs. Kendall could not but be gratified with the warmth of feeling displayed by her many admirers of both sexes.

The Lord Mayor, on Monday, opened a bazaar on behalf of the new buildings of the Great Northern Central Hospital in Holloway Road. Among the stall-holders were the Baroness Burdett-Coutts, Mrs. T. C. Murdoch, Mrs. Cowley Lambert, Mrs. Corry Wright, and Mrs. Marshall Lang. The inhabitants of North London, who have been warmly encouraged and aided by the Baroness and Mr. Burdett-Coutts, have made strenuous exertions on behalf of the new hospital during the last six or seven years. About twenty-five thousand pounds are still required to complete the building for 150 beds. The out-patients' department, which is now open, is in many respects a model of completeness and convenience. The three large wards also, as well as the domestic and administrative departments, which are now in use, are good examples of the modern methods of hospital construction, and will well repay a visit.

Lord Randolph Churchill, M.P., will preside at a special festival dinner to be held at the Hôtel Métropole, on May 12th next, in aid of a scheme for the consolidation and extension of St. Mary's Hospital. The object of the scheme is to acquire the piece of land which extends from the present hospital site to Praed Street, and upon which the houses Nos. 82 to 112, Praed Street, now stand. This will at once furnish space for the extension of the hospital—a work which cannot long be delayed if the charity is to provide adequately for the needs of the district—and, by giving it a frontage on the main street, will remove an obstacle which has prejudiced the progress of the charity from its foundation. The opportunity to acquire this land is now at the disposal of the governors, and may never again recur. Now, therefore, is the time for action to be taken. It is, however, a great work, and will involve a large expenditure, beside necessitating a material increase in the reliable income of the charity. To raise a fund for this purpose, and to enlist the aid of new friends in the good work of the hospital, are the objects for which this festival dinner is to be held, and in aid of which support is now solicited. The future well-being of the institution in a great measure depends upon the result of the efforts now being made, and it is therefore confidently hoped that the hospital will not seek in vain for friends at such an important crisis in its history. From personal knowledge of the value of this important charity we give the scheme our hearty endorsement.

Everybody should read the report of the Society for the Prevention of Cruelty to Children, which has just been issued. It is a duty to do so. We ought all to know for our guidance what kind of creatures untaught men and women can become. If anything can justify a man in thoroughly hooting other men the reading of this report will do so. The only thing which could fully satisfy the deep anger which the stories therein published arouse would be the extinction from the face of the earth of such monsters of cruelty as are here revealed in the light of day.

The Fitzroy Choral Society gave a concert to the in-patients of St. John's Hospital for Diseases of the Skin, Leicester Square, on Saturday evening. The concert was organised by Mr. Roper, the energetic honorary secretary of the choir, and was a great success. The programme was a very good one. Among the items that were encored were "Twickenham Ferry" (Miss Dally), "Anchored" (Mr. W. Birch), "Silver Rhine" (Miss L. Taylor and a delightful trio); "A Little Farm Well Tilled" (Mr. Lizard, Mr. Oliver—the conductor of the choir—and Mr. Birch.) Mr. R. H. Tickle, organist of St. Matthias' Church, Bethnal Green, accompanied the several songs and also played two charming pianoforte solos.

What can you do for a permanently invalided child for seven shillings a-week? Not much, we say, in London. Miss Bowditch, a clergyman's daughter, who has set up a home for invalid children at Coldash, near Newbury, in Berkshire, answers differently. About eighteen months ago she began with two little boys, suffering from hip-joint disease, and a single nurse to look after them. Within two months two more beds were added and filled; before the close of the year two more beds were added and filled; and in the spring of last year the numbers were increased to eight, and subsequently to twelve. The home is always full; some of the little ones have been there a year, others two, three, or six months, as the case may require. The nursing is entirely done by ladies, and the children are much with them. The institution is essentially a "home," not a "hospital." There is a cow, fowls, donkey, etc., all of which are pets, and fed by the children, who are out of doors as much as possible. Those well enough have gardens of their own, and the tending of these is a great interest. The surrounding country consists of open commons and pine woods, and the house stands on gravel soil, and has a good garden. For all this the minimum charge for the poor is 7s. a-week, but those who can afford it pay a larger sum. Miss Bowditch looks forward to the day when she will be able to enlarge the house to receive twenty patients, and has plans in preparation for this. The home is not supported by charity, though help is occasionally asked to enable a special case to remain longer in it. If its existence were better known it is probable that the demands on its space would be large, as many of those interested in invalid children would be glad to send them to a place where they would be sure to be taken care of, and where the terms are moderate. The only cases refused are infectious ones and babies under two years of age.

The following vacancies are announced this week, the amount of salary in each case being given after the name of the institution :—

**Resident Medical Officers.**

Croydon General Hospital. House Surgeon.—£100 per annum, with board, increasing to £170.
Hertford British Hospital, Paris. House Surgeon.
Hospi al for Diseases of the Throat, Golden Square. Resident Medical Officer.—£50 per annum, with board.
Newport and County Infirmary. House Surgeon.—£100 per annum, with board.
North Staffordshire Iofirmary House Physician.—£100, with board, etc.
North-West London Hospital, Kentish Town Road. Senior Resident Medical Officer.
Parish of Lochs, Stornoway. Medical Officer.—£140 per annum, with house.
Royal Hospital for Diseases of the Chest, City Road. Junior House Physician.—£50 per annum, with board.
Warwick County Lunatic Asylum, Hatton, near Warwick. Assistant Medical Officer.—£100 per annum, with board.

**Non-resident Medical Officers.**

Ancoats Hospital, Manchester. Junior Visiting Surgeon.—£60 per annum.
Bloomsbury Dispensary. Physician.
Hospital for Epilepsy and Paralysis, 32, Portland Terrace, Regent's Park. Assistant Physician and Registrar.
London Temperance Hospital. Registrar and Chloroformist.—£50 per annum.
National Lying in Hospital, Holles Street, Dublin. Two Assistants to Master.
Parish of Barnet, etc. Medical Officer of Health.—£443 per annum.
Royal Free Hospital, Gray's Inn Road. Assistant Physician.
Royal Free Hospital, Gray's Inn Road. Assistant Surgeon.

"The hospital," says the Bishop of Peterborough, "is the crown and glory of English charitable institutions, fulfilling more than anything else the ideal of mercy, which is 'twice blessed.' It blesses the poor, who are treated in its wards with the utmost skill and care ; and it blesses the rich, to whose bedside it brings the training, the science, and the skill which can only be acquired in the wards of a hospital."

Dr. John McIntyre, of Odiham, has presented to the students of Glasgow University, which is his *Alma Mater*, a splendid Union Club building, erected in the University grounds, and containing a library, a hall for debates, a dining-room, committee-rooms, and all other arrangements necessary for a club-house. The building has cost £5,000, and at Dr. McIntyre's request the benefits of it are not to be confined to the University students, but are to extend, under certain regulations, to those of the Extra-Mural Medical Schools.

A new departure is to be noted in the Sussex County Hospital. On the site of the museum large detached wards have been erected, which are meant to be used for violent patients, or for those suffering from erysipelas, etc. The wards on the top floor constitute almost a separate establishment, having a separate entrance and staircase, as well as bath-room and offices, so that complete isolation of infectious cases is secured. The wards are bright and cheerful, and all the appointments are of the newest and most approved kind. Altogether the addition will be a great advantage to the hospital, and reflect great credit on Mr. Scott, the architect.

The first annual meeting of the Native Races and the Liquor Traffic United Committee was held on the 4th in Princes' Hall, Piccadilly, the Bishop of Sodor and Man in the chair. This committee is formed of gentlemen representing individuals and socities interested in missionary and temperance work, and as yet its work has been chiefly one of investigation and vigilance. Several tokens of encouragement in the work has been received, among others, the debate in Parliament on the Liquor Traffic in India, the Islands of the Western Pacific and Africa, as well as the recent action of the Cape Government in regard to the restrictions of the trade. Funds are, however, urgently needed, and should be sent to the Hon. Secretary, the Rev. J. Grant Mills, at 127, Palace Chambers, Westminster, S.W.

The year commenced with a debit but ended with a credit-balance. Such is the substance of the financial statement of the Richmond Hospital for 1887. The debt amounted to £125 19*s*. 1*d*. at the beginning of the year; the credit-balance to £163 7*s*. 5*d*. at the end of it. The scale was turned by two legacies of £200 and £100 respectively ; but as those were not very large amounts, the management deserve credit for the financial improvement. The working-class do well at Richmond. Last year they contributed no less than £313 11*s*. 7*d*. to the hospital funds. Admiral Stopford and the committee and officials over whom he presides are to be congratulated on the good account they have given of the year's stewardship.

We desire to draw the attention of our readers to the claims on their sympathy and support of the Cancer Hospital, Fulham Road, Brompton, S.W., which was founded specially for the treatment of the poor afflicted with this terrible disease, who are admitted free, from all parts of the United Kingdom, without requiring letters of recommendation. During the year 1887 there were 703 new cases admitted as in-patients, and 1,012 as out-patients, and the number of attendances in the out-patient department shows a large increase over previous years. The nature of the disease requires a most generous diet, as well as treatment of the most expensive kind ; and to meet the growing demand upon their resources the committee urgently appeal for increased funds to enable them to satisfactorily cope with their liabilities. The ordinary expenditure of the hospital is some £3,000 a year more than the reliable income, and to meet this deficit new annual subscriptions and donations are earnestly solicited, and will be thankfully received by the bankers, Messrs. Coutts and Co., 59, Strand ; or by the secretary, Mr. W. H. Hughes, at the hospital, Fulham Road, S.W.

Misunderstandings and quarrels at hospitals are always painful, and often quite unnecessary. The Dorset County Hospital at the present moment seems to be in a condition of acute tension about matters which ought to be capable of settlement on reasonable lines. There is apparently a disagreement between the doctors and the matron. The committee, it appears, take the side of the matron, whilst many of the governors hold with the doctors. At this distance, and without special information, it is impossible for us to speak with very much confidence ; but, so far as published reports enable us to form a judgment, we incline to the opinion that the committee are somewhat in error. One point in dispute, and perhaps the principal point, is that the committee insist upon the matron going round the wards with each medical man, and receiving instructions from him as to the nursing of each patient. Those instructions the matron in her turn gives to the nurse in charge of the case. The matron thus acts as an intermediary between the doctor and the nurse. If our information be correct, and this be a true statement of the case, we cannot but express our disapproval of the arrangement. In a small cottage hospital, where the matron may be the only trained nurse and the only intelligent woman in the place, such a custom may be desirable and indeed inevitable; but in a hospital of any magnitude, where there must be at least one capable nurse to each ward, and where the matron cannot but have abundance of occupation elsewhere, it is not only undesirable, but injurious. Under such circumstances the nursing must be less well performed, and other departments of the matron's work cannot but also suffer. The proper course always to take is to employ nurses who are fit for responsibility, and then to put responsibility upon them. The rule must be that the nurse actually in charge of the case receives her instructions directly from the medical attendant. Where such a rule is departed from, the departure can only be justified by exceptional circumstances rendering it impossible for the rule to be observed.

